# RHPS4 G-Quadruplex Ligand Induces Anti-Proliferative Effects in Brain Tumor Cells

**DOI:** 10.1371/journal.pone.0086187

**Published:** 2014-01-15

**Authors:** Sunil Lagah, I-Li Tan, Priya Radhakrishnan, Robert A. Hirst, Jennifer H. Ward, Chris O’Callaghan, Stuart J. Smith, Malcolm F. G. Stevens, Richard G. Grundy, Ruman Rahman

**Affiliations:** 1 Children’s Brain Tumour Research Centre, School of Clinical Sciences, University of Nottingham, Nottingham, United Kingdom; 2 Department of Infection, Immunity and Inflammation, Leicester Royal Infirmary, University of Leicester, Leicester, United Kingdom; 3 Department of Respiratory Medicine, Portex Unit, Institute of Child Health, University College London and Great Ormond Street Hospital, London, United Kingdom; 4 School of Pharmacy, Centre for Biomolecular Sciences, University of Nottingham, Nottingham, United Kingdom; University of Pécs Medical School, Hungary

## Abstract

**Background:**

Telomeric 3′ overhangs can fold into a four-stranded DNA structure termed G-quadruplex (G4), a formation which inhibits telomerase. As telomerase activation is crucial for telomere maintenance in most cancer cells, several classes of G4 ligands have been designed to directly disrupt telomeric structure.

**Methods:**

We exposed brain tumor cells to the G4 ligand 3,11-difluoro-6,8,13-trimethyl-8*H*-quino[4,3,2-*kl*]acridinium methosulfate (RHPS4) and investigated proliferation, cell cycle dynamics, telomere length, telomerase activity and activated c-Myc levels.

**Results:**

Although all cell lines tested were sensitive to RHPS4, PFSK-1 central nervous system primitive neuroectodermal cells, DAOY medulloblastoma cells and U87 glioblastoma cells exhibited up to 30-fold increased sensitivity compared to KNS42 glioblastoma, C6 glioma and Res196 ependymoma cells. An increased proportion of S-phase cells were observed in medulloblastoma and high grade glioma cells whilst CNS PNET cells showed an increased proportion of G1-phase cells. RHPS4-induced phenotypes were concomitant with telomerase inhibition, manifested in a telomere length-independent manner and not associated with activated c-Myc levels. However, anti-proliferative effects were also observed in normal neural/endothelial cells *in vitro* and *ex vivo*.

**Conclusion:**

This study warrants *in vivo* validation of RHPS4 and alternative G4 ligands as potential anti-cancer agents for brain tumors but highlights the consideration of dose-limiting tissue toxicities.

## Introduction

Human telomeres are repetitive TTAGGG sequences located on the ends of chromosomes allowing cells to distinguish between natural chromosome ends and double-strand DNA breaks [1], [2]. The perpetual maintenance of telomeric DNA allows tumor cells to possess unlimited replicative potential, one of the hallmarks of cancer [3]. Activated telomerase maintains telomere length homeostasis in ∼85% of human cancers [4] justifying the numerous anti-cancer strategies targeting components of the telomerase holoenzyme [5], [6], [7], [8], [9], [10], [11], [12]. However, such approaches require telomeres on one or more chromosome ends to be critically eroded before any anti-cancer phenotype is observed [13]. An alternate approach to cause both shortening of telomeres and telomere uncapping is the use of G-quadruplex (G4) ligands. As telomerase requires the 3′ telomeric end to be in a single-stranded configuration, sequestering of the telomere in a four-stranded structure by small molecules that can compete with telomere-associated proteins, inhibits the binding of telomerase to telomere ends. The resulting loss of telomere maintenance precedes activation of a DNA damage response and growth arrest [14]. Many chemical classes of G4 ligands have been described which reduce the growth of various cancer cell lines *in vitro* [15], [16], [17], [18], [19] and exhibited antitumor activity *in vivo* [20], [21]. However, direct evidence of G4-mediated telomerase inhibition as a proximal event is minimal due to a lack of reliable *in vitro* telomerase assays. The claim of telomerase inhibition in many studies could be erroneous due to the inhibition of Taq polymerase by G4 ligands [17], [22]. More recent re-evaluations of telomerase inhibition by G4 ligands support this claim [22], [23], [24]. Although any G4 ligand that can inhibit the replication of TTAGGG_n_ by Taq polymerase will likely also inhibit telomerase, IC_50_ values determined from such a telomerase activity assay are likely to be incorrect. There is therefore a need for more accurate telomerase detection methods that may circumvent the requirement of Taq polymerases. In addition to preventing telomerase access to the telomere substrate, G4 ligands can exert anti-cancer effects as a result of uncapped telomeres due to the loss of binding of telomeric proteins such as POT1, TRF 1 and TRF2. G4 ligand induced effects can further be potentiated through stabilization of G-quadruplexes at non-telomeric G-rich loci, particularly promoter regions of oncogenes such as c-Myc [25], [26], [27], [28].

Pentacyclic 3,11-difluoro-6,8,13-trimethyl-8*H*-quino[4,3,2-*kl*]acridinium methosulfate (RHPS4) was part of a series of acridinium salts that were synthesized at the University of Nottingham (Nottingham, UK) which showed preference for binding to and stabilizing, G4 DNA isoforms over DNA duplexes [29], [30], [31], [32]. Treatment of various cancer cell lines and tumor xenografts revealed that RHPS4 was a potent telomerase inhibitor at submicromolar concentrations, caused irreversible proliferation arrest after long-term culture at non-cytotoxic concentrations and exhibited antitumoral activity *in vivo* [17], [29], [33]. However when used at higher doses, RHPS4 triggered short-term apoptosis/senescence in human melanoma cells [17] and a potent DNA damage response at the telomeres of transformed human fibroblasts, melanoma cells and uterine cancer cells, characterized by the appearance of the phosphorylated DNA damage response factors γ-H2AX, RAD17 and 53BPI. This phenotype however, was antagonized by overexpression of the telomere binding proteins POT1 and TRF1 [14], [34].

Here we investigated G4 ligand-mediated anti-tumor effects on malignant childhood and adult brain cancer cells *in vitro* using the pentacyclic acridine RHPS4 as proof-of-concept and further assessed toxicity of RHPS4 *in vitro* and in functional *ex vivo* assays.

## Materials and Methods

### Cell Lines

PFSK-1 (pediatric central nervous system primitive neuroectodermal tumor (CNS PNET)), DAOY (pediatric medulloblastoma), C6 (rat glioma) and U87 (adult glioblastoma) cell lines were obtained from American Type Culture Collection (ATCC, Manassas, VA, USA). The GB-1 line (reclassified as pediatric grade III mixed glioneuronal), was derived at the University of Birmingham, UK and previously reported by us [35]. KNS42 (pediatric glioblastoma) was a kind gift from Dr. Chris Jones at the Institute of Cancer Research, London and previously isolated and characterized [36]. Res196 (pediatric ependymoma) was a kind gift from Dr. Michael Bobola at Seattle Children’s Hospital Research Institute [37]. C17.2 neural progenitor cells isolated from neonatal mouse cerebellar cortex and immortalized with v-Myc have been previously described [38]. Human brain microvascular brain endothelial cells (HBMEC) were a kind gift from Dr. Naveed Khan, University of Nottingham [39].

### Cell Culture and Drug Preparation

Cells were cultured in Dulbecco’s modified Eagle’s medium (DMEM) (Sigma, UK) (DAOY, C6, GB-1, U87 and C17.2), RPMI-1640 (Sigma, UK) (PFSK-1) or DMEM/F12 (Sigma, UK) (KNS42 and Res196), supplemented with 10% fetal bovine serum (FBS) (or 10% FBS/5% horse serum (C17.2)) (PAA Labs, UK). HBMEC cells were cultured in RPMI-1640 media as previously described but supplemented with 20% fetal bovine serum and 1% MEM vitamins (Invitrogen, UK).

### Proliferation Assay and Drug Exposure

Cells were seeded at a density of 5×10^4^ cells per well of a 24-well plate, 24 hours prior to 0.5–50.0 µM RHPS4 exposure for 72 hours. Alamar Blue assay (Invitrogen, UK) was conducted according to the manufacturer guidelines and fluorescence emission measured at 585 nm using a plate reader (Tecan, Switzerland). Percentage viability was calculated related to vehicle-only treated controls. Qualitative images of RHPS4-treated brain tumor cells were taken using a standard light microscope (Leica, UK). IC_50_ values refer to the concentration at which the tumor population viability is 50% that of the corresponding untreated tumor population.

### Cell Cycle Analyses

RHPS4-treated cells were fixed with cold ethanol, washed with cold PBS (with 0.1% BSA, 0.1% Tween) and stained with 20 µg propidium iodide for 20 minutes in the dark and at room temperature. Cell cycle analysis was conducted using a Coulter FC500 flow cytometer and analyzed using WinMDI2.8 and Cylchred software. Four independent experiments were conducted for all samples with the mean percentage of each fraction presented.

### Telomere Restriction Fragment (TRF) Length Assay

The TRF assay determines mean telomere length in a population of cells or in tissue. Mean telomere length was determined using the TeloTAGGG kit according to the manufacturer guidelines (Roche, Burgess Hill, UK). Briefly, 3 µg genomic DNA per sample was digested using a frequent cutter restriction enzyme mix, leaving undigested telomeric DNA, which was resolved on 0.8% agarose gel. Blots were transferred to a nylon membrane, probed with a telomere-specific probe and exposed to film. The average TRF length was determined by comparing telomere smear and intensity signals relative to a molecular weight standard using ImageQuant version 5.1 software (GE Healthcare, Buckinghamshire, UK).

### Telomere Repeat Amplification Protocol (TRAP) Assay

Telomerase activity was analyzed using the TRAPeze telomerase detection kit (Millipore, Hertfordshire, UK). Standard TRAP assays were conducted using 100–500 ng protein according to the manufacturer guidelines and visualized with a Fujifilm FLA-2000 phosphoimager (Amersham Biosciences, Buckinghamshire, UK). For pre-extension modified TRAP assays, RHPS4 was added to cell-free lysates after a 5 minutes telomere extension step (to generate a telomere product of at least four hexameric repeats) but prior to an additional 25 minutes telomere extension. For post-extension modified TRAP assays, RHPS4 was added to cell-free lysates immediately upon completion of telomere extension. To eliminate RHPS4 prior to the PCR stage, telomere extended products were first ethanol precipitated after phenol/chloroform. Quantitative TRAP assays were conducted using the TRAPeze XL telomerase detection kit according to manufacturer guidelines (Millipore, Hertfordshire, UK). The TRAP assay was conducted three times using independent RHPS4-treated TS oligonucleotides.

### c-Myc Transcription Factor Assay

Activation of c-Myc was analyzed using the TransAM™ c-Myc transcription factor assay kit (Active Motif, UK). TransAM® Kits are sensitive, non-radioactive transcription factor ELISA kits that facilitate the study of transcription factor activation in mammalian tissue and cell extracts. Nuclear extracts were isolated from brain tumor cells treated with RHPS4 for 72 hours using the Nuclear Extract kit (Active Motif, UK) and 2 µg of nuclear extract used in the c-Myc transcription factor assay according to the manufacturer guidelines. Jurkat nuclear extract was used as a positive control. Three independently-derived nuclear extracts were used for each sample.

### c-Myc Quantitative Reverse-transcriptase PCR

Total RNA was extracted from RHPS$-treated cell pellets using the mirVanaTM miRNA Isolation kit (Applied Biosystems, Carlsbad, CA, USA). cDNA synthesis was carried out by incubating 500 ng RNA with 200 U reverse transcriptase (Fermentas, St. Leon-Rot, Germany) at 42°C for 1 hour. PCR reactions were carried out using 1Q Custom SYBR Green Supermix (Bio-Rad, Hercules, CA, USA) and 100 nM forward and reverse primers (human c-Myc forward primer ACTCAGTCTGGGTGGAAGGT, reverse primer CGTATACTTGGAGAGCGCGT; rat c-Myc forward primer CTGTACGCCCAAACGCAAAA, reverse primer TTAGTCACCGCAGGTGGAAC). The CFX96 real time PCR machine (Bio-Rad) was used. PCR conditions were 95°C for 10 minutes followed by 40 cycles of 95°C for 30 seconds, 60°C for 1 minute and 72°C for 1 minute. Data was normalized using GAPDH (forward primer CGCTCTCCAGAACATCATCC, reverse primer GGAGATTCAGTGTGGTGG).

### Ependymal Cell Culture and Cilia Function *ex vivo*

Primary ependymal cells were grown as described previously in [40]. Ciliated adherent ependymal colonies were cultured with or without RHPS4 (3 µM or 30 µM). To determine cilia beat frequency (CBF) and cilia tip distance travelled, cultured cells were placed in a humidified incubation chamber (37°C) and observed using an inverted microscope (TxU, Nikon, UK). Beating cilia were recorded using a digital high-speed video camera (X4, Lake Image Systems, UK) at a rate of 500 frames per second. Each time-point represents the measurement of CBF and tip distance at 5 regions of interest (ROI) from 3 ciliated cells (5×3 = 15 readings per time point). The data are from four separate primary cultures from four rat brains. Calculation of CBF (Hz): 500 (no. frames per second)/5 (frames elapsed for five ciliary beat cycles)×5 (conversion per beat cycle). The captured video sequences were played back at a slow rate which allowed determination of the distance travelled by cilia tips within the power stroke of the beat cycle.

### Statistical Analyses

SPSSv16 was used for performing all statistical analyses. Independent-sample t-tests with 95% confidence intervals were used to compare the viability of RHPS4-treated versus untreated cells and to assess c-Myc activation levels with or without RHPS4 exposure. Data is presented as the average relative fluorescence units or absorbance respectively, of three independent experiments with error bars indicating standard error of the mean values. Correlation between results from the Alamar Blue proliferation assay and TRF length assays was determined by a Pearson’s correlation test. P-values less than or equal to 0.05 were deemed statistically significant throughout. One-way ANOVA analyses was conducted to establish the variance between untreated and drug-treated samples in the ciliated ependymal cell toxicity assay and the Tukey HSD sub-group test was used to establish significant differences between groups at specific time intervals. One-way ANOVA analyses was used to determine significance between drug-treated and untreated samples with respect to each cell cycle fraction, where a p-value of less than or equal to 0.05 was deemed to represent a significant increase or decrease in the respective fraction.

## Results

### Acute Exposure to RHPS4 Results in Dose-dependent Inhibition of Growth in Brain Tumor Cells

**Figure 1:**
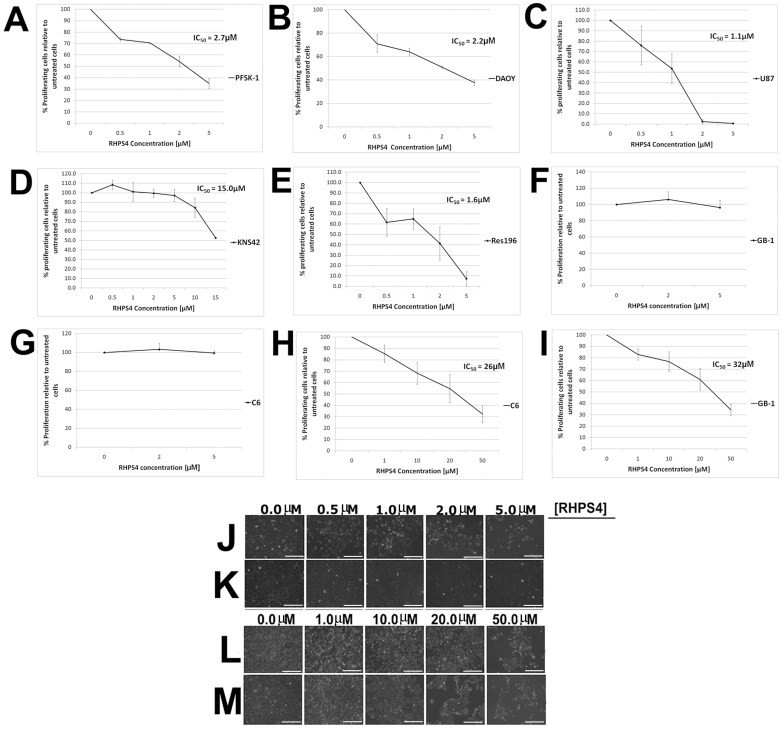
Acute RHPS4 exposure inhibits proliferation of high grade brain tumor cells *in vitro*. Proliferation of tumor cells was impaired in malignant brain tumor cells after acute 72 hours exposure to RHPS4. (A–C) PFSK-1, DAOY, U87 and (E) Res196 cells exhibited IC_50_ values of 2.7, 2.2, 1.1, and 1.6 µM respectively when 0.5–5.0 µM RHPS4 was used, representing a significant inhibition of cell proliferation (p≤0.05 for each drug concentration versus untreated). (D, F–G) Within this concentration range, KNS42, C6 and GB-1 cells were resistant to RHPS4. (H–I) At higher concentrations of RHPS4 exposure C6 and GB-1 cells exhibited IC_50_ values of 26 µM and 32 µM respectively, representing a significant inhibition of cell proliferation (p≤0.05 for each drug concentration versus untreated). Error bars indicate standard error from three independent experiments. (J–M) Light microscopy of PFSK-1, DAOY, C6 and GB-1 cells showing a marked reduction in cellular density after RHPS4 exposure. *Magnifications, x20; Scale bar = 25* *µm*.

CNS PNET, medulloblastoma, high grade glioma and ependymoma cells were treated with RHPS4 for an acute 72 hour period at an initial concentration range of 0.5–5.0 µM, followed by a concentration range of 1.0–50.0 µM for relatively less sensitive cell lines. PFSK-1, DAOY, U87 and Res196 cells display a significant dose-dependent viability loss at 0.5–5.0 µM RHPS4 with half maximal inhibitory concentration (IC_50_) values of 2.7, 2.2, 1.1 and 1.6 µM respectively (p≤0.05 for each drug concentration versus untreated). Approximately 2–40% of viable cells remained after the highest drug dose (5 µM) after 72 hours treatment (Figure 1A, C and E). Within this concentration range, KNS42, C6 and GB-1 cells are resistant to RHPS4 (Figure 1D and F, G). However at concentrations >10.0 µM, KNS42, C6 and GB-1 display a significant dose-dependent viability loss with IC_50_ values of 15.0, 26.0 and 32.0 µM respectively (p≤0.05 for each drug concentration versus untreated). Approximately 30–50% of viable cells remained after the highest drug dose after 72 hours treatment (Figure 1D and H, I). Light microscopy of RHPS4-treated PFSK-1, DAOY, C6 and GB-1 cells, qualitatively shows a dose-dependent viability loss with marked growth arrest at concentrations above 2.0 µM RHPS4 for PFSK-1 and DAOY cells and 20.0 µM RHPS4 for C6 and GB-1 cells after 72 hours treatment (Figure 1J, M). Non-acute concentrations of RHPS4 however (10-fold below that which resulted in an IC_50_ value in the acute regime), had no obvious effect on population doubling time in PFSK-1 and C6 cells after 23 and 36 days respectively, chosen as representative cell lines for differential RHPS4 sensitivity (Figure S1). These results provide proof-of-concept that several brain tumor cell types are dose-responsive to the RHPS4 G4 ligand *in vitro* with PFSK-1, DAOY, U87 and Res196 cells exhibiting 10 to30 fold greater sensitivity than KNS42, C6 and GB-1 cells. Further molecular and cellular characterizations were conducted using cell lines representative of the observed difference in RHPS4 sensitivity, with PFSK-1/DAOY representing relatively greater sensitivity to RHPS4 and C6/GB-1 representing relatively reduced sensitivity to RHPS4.

### Alterations in Cell Cycle Dynamics upon RHPS4 Exposure *in vitro* are Cell Line Dependent

**Figure 2:**
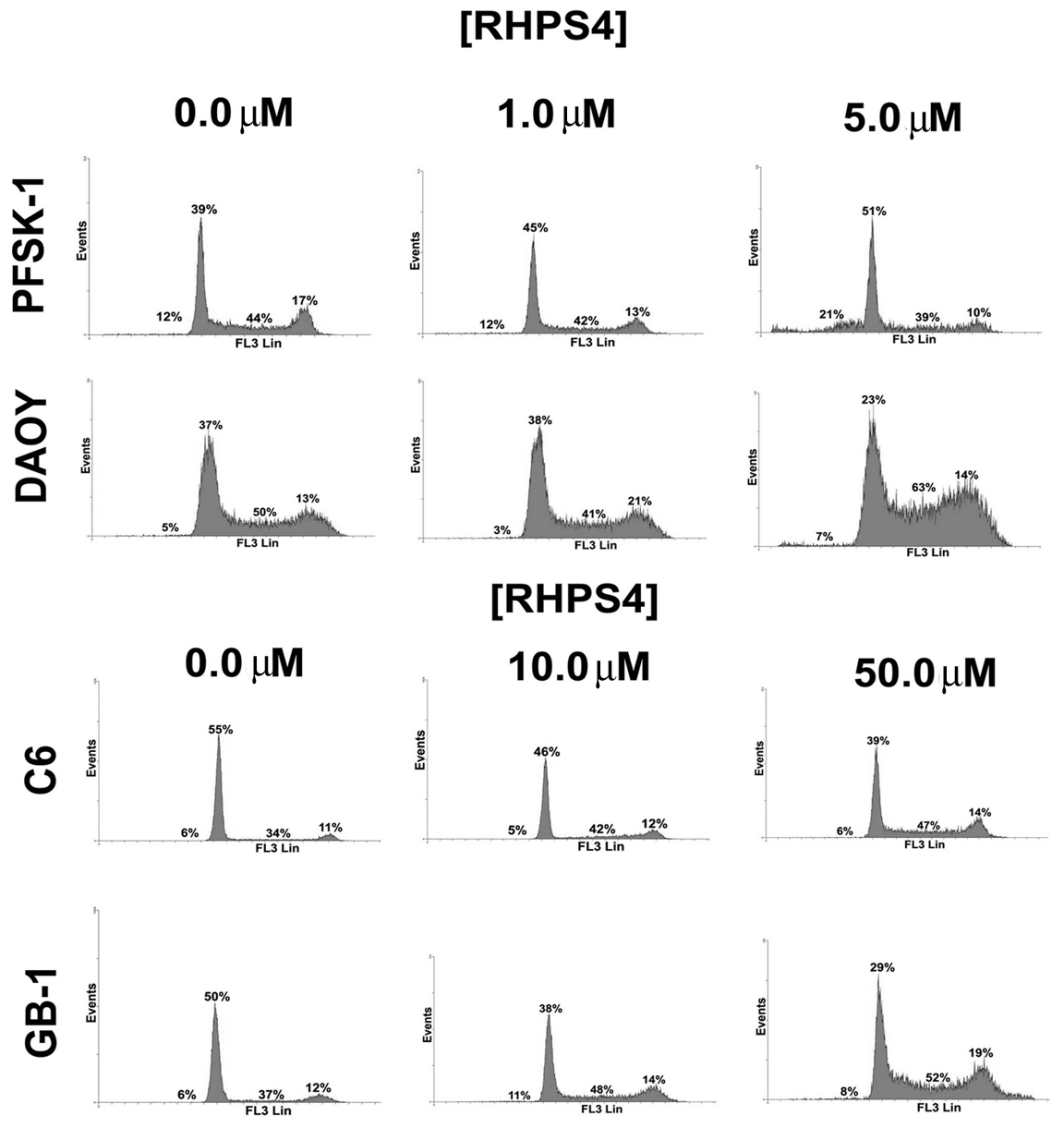
Acute RHPS4 exposure alters cell cycle dynamics of brain tumor cells *in vitro*. PFSK-1 cells exhibited a dose-dependent increase in the proportion of cells in G1-phase. In contrast DAOY, C6 and GB-1 cells exhibited a dose-dependent increase in the proportion of cells in S-phase. PFSK-1 further shows a moderate accompanying increase of sub-G0/1 cells at the higher RHPS4 concentration (5 µM). Percentages are the mean from three independent experiments. Asterisk denotes a significant difference relative to untreated cells.

Based upon IC_50_ values, cell lines were treated with appropriate RHPS4 concentrations for 72 hours and cell cycle profiles analyzed. PFSK-1 cells showed a significant (F_2,9_ = 6.97, p = 0.015) increase in the proportion of cells in G1-phase which was dose-dependent, with a moderate but significant (F_2,9_ = 4.752, p = 0.039) increase of cells in the sub-G0/1 stage at the higher RHPS4 concentration (5 µM). In contrast DAOY, C6 and GB-1 showed a broadly dose-dependent significant (F_2,9_ = 10.35, p = 0.005; F_2,9_ = 4.26, p = 0.05; and F_2,9_ = 6.24, p = 0.02 respectively) increase in the proportion of cells in S-phase, with a slight accompanying increase of DAOY sub-G0/1 cells observed at the higher RHPS4 concentration (5 µM), although the latter did not reach statistical significance (Figure 2). Although an increase in the G1/S fraction suggests impaired proliferation of brain tumor cells relative to untreated cells, drug-treated cells were observed to recover and proliferate after withdrawal of RHPS4, presumably due to the presence of RHPS-resistant clones (within the drug concentrations used in these experiments) (Figure S2).

### RHPS4-mediated Short-term Anti-cancer Effects *in vitro* and Association with Telomere Length

**Figure 3:**
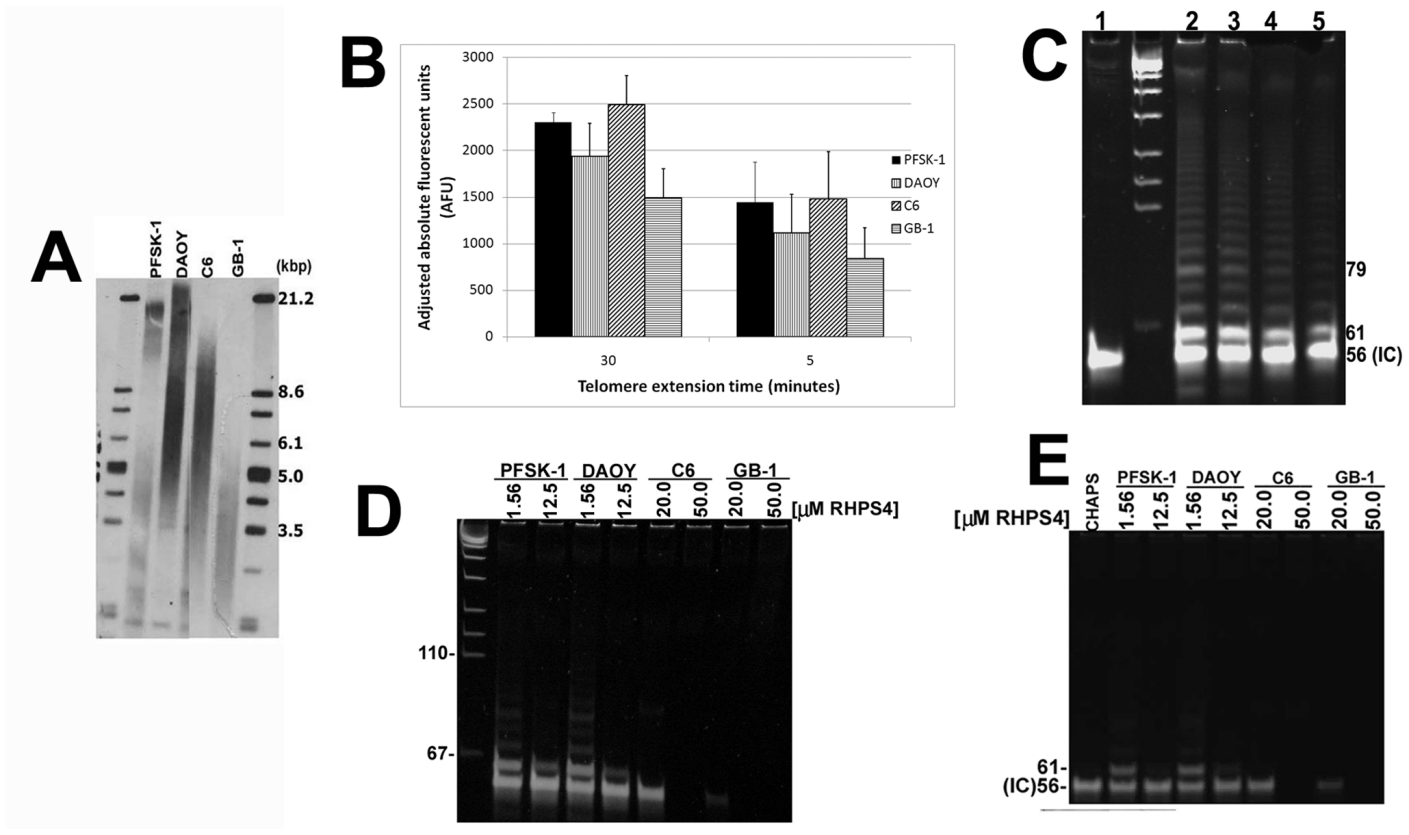
Telomere length measurement and RHPS4-mediated inhibition of Taq polymerase *in vitro*. (A) Mean TRF lengths for PFSK-1/DAOY cells (3.8 kb/7.8 kb) and C6/GB-1 (7.5 kb/3.9 kb) cells were determined prior to RHPS4 exposure. (B) Quantitative TRAP assay showing telomerase-mediated telomere extension after 30 minutes (standard TRAP assay) or 5 minutes extension time in non-drug exposed cells. (C) PCR gel showing telomere extension products after 5 minutes extension time in non-drug exposed cells. *1, no lysate control; 2–5, PFSK-1, DAOY, C6 and GB-1*. (D) PFSK-1 and DAOY showed low levels of telomerase activity at low RHPS4 concentrations and complete absence of activity at high RHPS4 concentrations, when RHPS4 was added pre-telomere extension. C6 and GB-1 showed complete absence of telomerase activity at both RHPS4 concentrations analyzed. (E) Telomerase activity was absent in all cell lines and at both RHPS4 concentrations when RHPS4 was added post-telomere extension. *CHAPS, CHAPS buffer only no lysate control; IC, internal control 61-bp telomere substrate oligonucleotide*.

As folding of the single-strand telomeric substrate into a four-stranded quadruplex structure inhibits the catalytic activity of telomerase [41], it is plausible that G4 stabilization results in telomerase inhibition proceeded by telomere shortening as a consequence. In this scenario, growth arrest is predicted to be directly related to initial mean telomere length. Therefore we hypothesized that the 10 to15 fold decreased sensitivity of C6 and GB-1 glioma cells treated with RHPS4 (compared to PFSK-1 and DAOY cells) is inversely proportional to mean telomere length. PFSK-1 and DAOY exhibited mean TRF lengths of 3.8 kb and 7.8 kb, respectively, whilst C6 and GB-1 glioma lines exhibited mean TRF lengths of 7.5 kb and 3.9 kb respectively (Figure 3A). Although no significant correlation was evident between 72 hour RHPS4 sensitivity and mean telomere length using representative tumor lines (Pearson’s coefficient r = −0.141, p<0.86), it is plausible that correlation with telomere length would be observed when considering RHPS4 sensitivities after >72 hour exposure within these cell lines.

### RHPS4 Inhibits Taq Polymerase in Cell-free Telomerase Activity Assays

To assess the effects of G4 stabilization directly at the telomere substrate, we introduced RHPS4 directly into cell-free TRAP assays using drug-treated protein/RNA lysates in order to discriminate whether RHPS4-mediated inhibition of telomerase excludes inhibition of Taq polymerase by this G4 ligand. A 5 minute telomere extension period using a telomere oligonucleotide substrate, permitted the synthesis of the minimum four hexameric TTAGGG telomere repeats required for G4 ligands to stabilize a four-stranded DNA structure (Figure 3B, C), prior to an additional 25 minutes telomere extension [23]. PFSK-1 and DAOY showed low levels of telomerase activity at low RHPS4 concentrations and complete absence of telomerase activity at high RHPS4 concentrations. Similarly C6 and GB-1 showed complete absence of telomerase activity at both RHPS4 concentrations analyzed (Figure 3D). However when RHPS4 was introduced to cell-free lysates immediately upon completion of 30 minutes telomere elongation but prior to the PCR amplification stage of the TRAP assay, telomerase activity was also absent and at all concentrations of drug analyzed (Figure 3E). Our results strongly indicate that RHPS4 inhibits Taq polymerase during the PCR stage of the TRAP assay and therefore RHPS4-mediated inhibition of telomerase activity cannot be determined when the ligand is introduced either prior to or immediately after, telomere extension.

### RHPS4 Inhibits Telomerase Activity in TRAP Assays Using Purified Telomere Products

**Figure 4:**
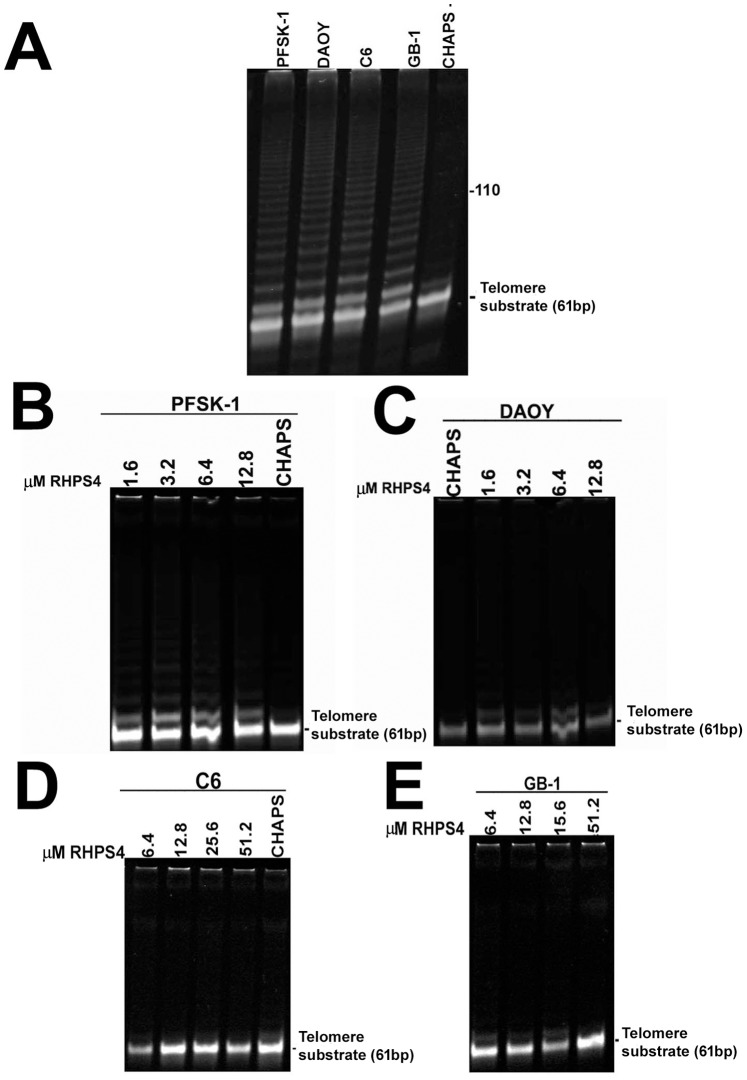
Acute RHPS4 exposure is associated with telomerase inhibition in brain tumor cells *in vitro*. (A) TRAP assay using ethanol-precipitated telomere extended DNA products after 30 minutes extension in non-drug treated brain tumor cells. High levels of telomerase activity are observed in each cell line. (B–D) TRAP assays in RHPS4-treated brain tumor lysates reveals complete telomerase inhibition in all cell lines at each drug concentration. 0.1 µg of total protein lysate was loaded per well in each TRAP assay. *CHAPS, CHAPS buffer only no lysate control; TS, telomere substrate internal control 61-bp oligonucleotide.*

Prior to PCR amplification stage, DNA extraction of elongated telomere fragments via ethanol precipitation was conducted to eliminate RHPS4 from telomere extension products. High telomerase activity was observed in all untreated cell lines after extracted telomere extended PCR products were resolved on acrylamide gels (Figure 4A). A drug concentration range in accordance to our previously established IC_50_ values (Figure 1) was used for the direct introduction of RHPS4 into the cell-free TRAP assay prior to purification of telomere extension products (1.6–12.8 µM for PFSK-1/DAOY; 6.4–51.2 µM for C6/GB-1). Substantial telomerase inhibition was observed in PFSK-1 cells with only very weak telomerase activity at each RHPS4 concentration (Figure 4B). Complete telomerase inhibition was observed in DAOY, C6 and GB-1 cells and at each drug concentration (Figure 4C, D, E). These results indicate that the presence of RHPS4 in a mixture containing cell-free brain tumor lysates and a telomere substrate oligonucleotide, results in a clear abrogation of telomerase activity *in vitro*. This result suggests one plausible mechanism through which RHPS4 may exert anti-proliferative effects in brain tumor cells utilized in this study. We cannot however exclude the possibility of some RHPS4 molecules remaining bound to the telomere substrate after ethanol precipitation, thereby impeding hybridization with telomere-specific primers and resulting in an overestimation of telomerase inhibition.

### RHPS4 Sensitivity is not Associated with Activation of c-Myc

**Figure 5:**
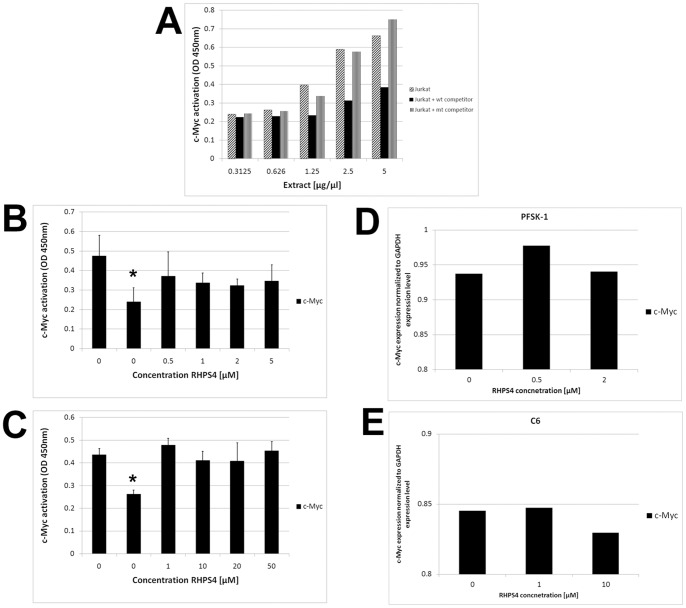
c-Myc activation is not associated with degree of RHPS4 sensitivity. (A) c-Myc transcription factor assay. Jurkat cell nuclear extracts show activation and specificity of c-Myc proportional to concentration of extract analyzed and in the presence of wild-type or mutant competitor. (B–C) No significant difference in c-Myc activation was observed between untreated PFSK-1 or C6 cells and RHPS4-treated cells. Asterisk denotes significant reduction in c-Myc levels when either PFSK-1 or C6 untreated cells were exposed to a wild-type oligonucleotide competitor (p≤0.05). (D–E) c-Myc quantitative reverse transcriptase PCR. No difference in PFSK-1 or C6 c-Myc gene expression was observed between representative RHPS4-treated cells and untreated cells.

To investigate whether other G-rich genomic sequences are susceptible to RHPS4, activated c-Myc protein levels were assessed upon exposure to RHPS4 using the TransAM™ c-Myc assay. The c-Myc protein was chosen on the basis that deregulation of c-Myc has been implicated in the origin of diverse human cancers and the c-Myc gene contains a G-rich promoter sequence [42], [43]. Jurkat tumor cell nuclear extracts show activation of c-Myc proportional to concentration of extract analyzed verifying the sensitivity of the TransAM™ c-Myc assay. A wild-type consensus oligonucleotide competitively binds to c-Myc, whereas a mutant oligonucleotide has no distinct effect, collectively demonstrating the specificity of the assay (Figure 5A). PFSK-1 and C6 cells were selected for representative analyses as these lines differed in RHPS4 sensitivity by ∼10-fold (Figure 1A and C). PFSK-1 cells treated with RHPS4 did not show a significant reduction in c-Myc activation at all drug concentrations analyzed relative to untreated vehicle-only controls (Figure 5B) (p≤0.05). Similarly, c-Myc activation levels at each RHPS4 concentration (1.0–50.0 µM) were comparable to untreated controls in C6 cells (Figure 5C) (p≤0.76). In both PFKS-1 and C6 cells, specificity for c-Myc expression was confirmed by addition of the wild-type oligonucleotide competitor during the assay; c-Myc expression levels were significantly reduced when this inhibitor was added to untreated cells (Figure 5B, C; p≤0.02). In addition, no reduction in c-Myc gene expression levels as determined by quantitative reverse transcriptase PCR, were observed in either PFSK-1 or C6 RHPS4-treated cells relative to untreated cells (Figure 5D, E). These results collectively suggest that RHPS4 sensitivity is not directly associated with downregulated c-Myc levels in brain tumor cells *in vitro*.

### Normal Neural and Endothelial Cells are Sensitive to RHPS4 Exposure *in vitro* and *ex vivo*

**Figure 6:**
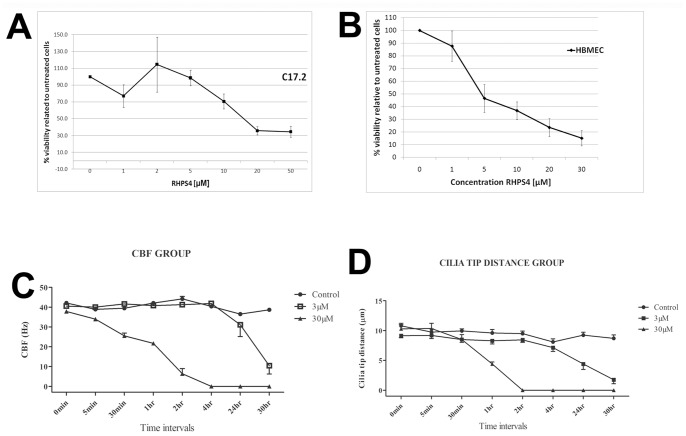
RHPS4 sensitivity in normal neural and endothelial cells *in vitro* and *ex vivo*. (A) C17.2 cerebellar progenitor cells and (B) HBMEC endothelial cells are sensitive to RHPS4 with an IC_50_ of 15 µM and 5 µM respectively. (C) Primary rat ependymal *ex vivo* cultures exhibited functional impairment of ependymal CBF after 3 µM or 30 µM RHPS4 exposure (p≤0.01). (D) A significant reduction in cilia tip distance was observed after either 3 µM or 30 µM RHPS4 exposure (p≤0.01). Error bars represent standard error of the mean from four separate rat brains/experiments.

To assess potential adverse cellular toxicity upon RHPS4 treatment, mouse cerebellar progenitor cells and human brain endothelial cells were analyzed for viability after an acute 3-day exposure to RHPS4. Both cell lines were sensitive to RHPS4 under these conditions, with an IC_50_ of 15 µM and 5 µM respectively (Figure 6A, B). To better elucidate what effects RHPS4 may have on the functional capacity of neural cells, we utilized an ependymal culture system *ex vivo* as a measure of neural cell function based on ependymal cilia. Primary rat ependymal cultures were assessed for functional impairment of ependymal CBF and cilia tip distance after 3 µM or 30 µM RHPS4 exposure relative to untreated cultures. A significant difference in CBF was observed across the entire cohort at 1 hour, 24 hours and 30 hours post-treatment (p≤0.0001). By 30 hours post-treatment, both the 3 µM and 30 µM groups showed a significant reduction in CBF compared to the untreated group (p≤0.01) (Figure 6C). Cilia tip distance was also measured in ependymal cultures and similarly showed a significant difference across the entire cohort at 1 hour, 24 hours and 30 hours post-treatment (p≤0.0001). By 24 hours post-treatment, both the 3 µM and 30 µM group showed a significant reduction in cilia tip distance compared to the untreated group (p≤0.01). (Figure 6D). These experiments collectively show that neural and endothelial cells display dose-dependent sensitivity to RHPS4.

## Discussion

Here we present a rationale for using G4 ligands for the treatment of certain childhood and adult brain tumors using RHPS4 as proof-of-concept of this class of anti-tumor agent and mechanism of G4 ligand action.

Strategies to inhibit telomerase have typically focused on targeting the human telomerase reverse transcriptase (hTERT) and human telomerase RNA (hTR) components of the telomerase enzyme [44], [45], [46], [47]. Anti-cancer phenotypes resulting from this approach are observed when one or more telomeres become critically short due to an absence of telomerase-mediated telomere maintenance, manifesting in senescence or apoptosis. As a telomere length-dependent lag may impact on the time to growth arrest in malignant cells, direct disruption of telomere structure whereby proliferation is arrested rapidly is an attractive strategy. Our result is consistent with previous studies that failed to observe telomere reduction in tumor cells after RHPS4 exposure [17], [29]. However our data do not exclude the possibility that non-acute nanomolar doses of RHPS4 or >72 hour drug exposure may induce gradual telomere shortening associated ultimately with a senescent phenotype. In particular, G4 ligand treatment of telomerase-positive glioma cell lines resulted in telomere instability, cell cycle alterations and apoptosis in a telomere length-independent manner, with IC_50_ values similar to those presented in our data [27]. However, our data does not exclude the possibility that telomerase inhibition at a subset of critically short telomeres may be necessary for RHPS4-mediated anti-proliferative effects as we have assessed mean telomere length within each tumor cell line.

RHPS4-treated brain tumor cells analyzed in this study exhibit an increase in the proportion of cycling cells in G1 or S phase, suggesting growth arrest in either phase. The very low sub-G0/1 fraction in drug-treated cells indicates that only few cells display a late-stage cell death phenotype, consistent with our direct qualitative visualization of cells. This suggests that RHPS4 in this context primarily acts to inhibit growth, at least within the time-frame and drug concentrations of the experiments used here. Although the Alamar Blue assay does not discriminate between proliferating cells and growth arrested but viable cells, the lack of a marked sub-G0/1 population as determined by cell cycle analyses and lack of directly observable dead cells in culture flasks, indicates that RHPS4-mediated anti-cancer effects are due to impaired tumor cell growth. However, our data indicates that resistant tumor cells that survive treatment are able to proliferate and re-populate the tumor population *in vitro*.

Phenomena induced by acute exposure to G4 ligands are likely due to activation of DNA damage pathways and subsequent genomic catastrophe. Prolonged stabilization of a quadruplex structure may prevent protection of POT1 and TRF1 proteins from binding to 3′ telomeric overhangs and thereby sheltering chromosomal ends from DNA damage surveillance [25]. The ∼10-fold difference in RHPS4 sensitivity between PFSK-1/DAOY embryonal cells and C6/GB-1 glioma cells is plausibly due to competitive binding of RHPS4 and POT1 to the 3′ overhang. In this scenario, the less sensitive C6 and GB-1 cells may exhibit higher levels of POT-1 protein at telomeres. In support of this hypothesis, potent DNA damage response at telomeres upon RHPS4 exposure is antagonized by overexpression of POT1 or TRF2 [14], [28]. Such findings suggest that G4 ligands can induce relatively rapid telomere uncapping and may explain why reductions in telomere length are not always observed in cells exposed to these compounds. As a corollary, POT1 or TRF1 displacement from telomeres may serve as appropriate biomarkers of G4 ligand target modulation in clinical trials. It is important to note however that whether RHPS4 potency associates with telomere length may be dependent on the tumor cell type under investigation. A panel of 36 xenograft cell lines derived from several human tumor types showed a strong correlation between telomere length and RHPS4 sensitivity [48]. The careful consideration of non-telomeric routes to toxicity was highlighted in a recent study describing light-dependent oxidative stress response, rather than G-quadruplex binding, as the major route to toxicity [49].

Several previous reports have claimed direct evidence of telomerase inhibition upon exposure of tumor cells to various G4 ligands [17]. These cell-free telomerase activity studies incubated ligands with protein/RNA lysates prior to telomere extension and PCR amplification. As several G4 ligands have more recently been shown to inhibit Taq polymerase during the PCR process, these studies may erroneously report telomerase inhibition [22], [23], [24]. In our study, telomere extended products were first precipitated after G4 ligands were incubated with protein/RNA lysates in addition to a telomere oligonucleotide substrate, prior to PCR amplification. Therefore the absence of telomere products observed in TRAP gels indicates true RHPS4-induced telomerase inhibition. Complete abrogation of telomerase activity in a cell-free assay was observed at RHPS4 concentrations below that required for 50% growth inhibition in drug-treated cell cultures. However, although our findings demonstrate RHPS4-mediated inhibition of telomerase activity via stabilization of a telomere oligonucleotide, direct evidence of telomerase inhibition in a cellular context is required. It will be intriguing to test the effects of RHPS4 against brain tumor stem-like populations that exhibit high levels of telomerase activity and long telomeres relative to the tumor population as a whole. Indeed the G4 ligand Telomestatin impairs glioma stem cell survival and growth through disruption of the telomere G-quadruplex and inhibition of the c-Myb proto-oncogene [26]. As c-Myc is de-regulated in several tumors and can contribute to the transcriptional activation of the hTERT gene in tumor cells, RHPS4-mediated stabilization at the c-Myc promoter may exacerbate telomerase inhibition effects in the tumor cell due to down-regulation of hTERT [50], [51]. Additionally, lack of c-Myc amplification in supratentorial PNET tumor tissue and in the PFSK-1 cell line may be a contributing factor to sensitivity to RHPS4 as patients with other PNET tumors which exhibit overexpression or amplification of c-Myc, have an extremely poor prognosis with poor response to chemotherapy [52], [53]. The baseline c-Myc protein level as determined by the Trans-AM assay is significantly higher in PFSK-1 cells (p≤0.3), but C6 c-Myc expression level is as high as 92% that of PFSK-1 c-Myc levels. Therefore basal c-Myc levels are unlikely to contribute to the differences in possible G4 stabilization at the c-Myc promoter in these cells. However our observation that activated c-Myc levels are not significantly down-regulated in either RHPS4-treated PFSK-1 or C6 cells, suggests that RHPS4-mediated effects in this context are not due to stabilization of G-rich elements at the c-Myc promoter. This is contrasted by studies demonstrating that the G-rich region upstream of the P1 promoter of the c-Myc gene controls ∼90% of its transcriptional regulation and that G4 ligands can stabilize the c-Myc promoter quadruplex and disable c-Myc in childhood medulloblastoma cells *in vitro* [54], [55], [56].

As RHPS4 exerted dose-dependent viability loss in normal neural/endothelial cells and impaired neural function in ciliated ependymal cells, our findings highlight the need for rigorous consideration of dose-limiting tissue toxicities when using G4 ligands for the targeting of cancer cells and/or G4 ligands with greater specificity for the binding to telomere substrates. Certain tumor types may be more amenable to this mode of therapy, where anti-cancer effects occur within a therapeutic window that results in acceptable toxicities to healthy tissue. Moreover non-acute repeated dosing of G4 ligands may be a more feasible strategy in the clinic. As several G4 ligands have been extensively studied in laboratory-based and pre-clinical studies, it will be important to test a broad range of these ligands specifically for brain tumor efficacy and neural toxicities using *in vitro* and *in vivo* strategies, prior to consideration in early phase patient trials.

## Conclusions

To our knowledge, this is the first report of RHPS4-induced viability loss in brain tumor cells. We have evaluated the poly-pharmacophoric nature of RHPS4 and shown that impairment of tumor cell viability is a proximal event and likely associated with G1/S phase arrest. We further suggest that RHPS4 sensitivity is not attributable to G-rich c-Myc promoter G-quadruplex but we cannot rule out stabilization at other non-telomeric loci.However, our data suggests the presence of RHPS4-resistant cells and direct evidence of telomerase inhibition in a cellular context is required. Our findings highlight confounds that need to be addressed for the consideration of G4 ligands as potential pharmacologic agents for the treatment of childhood and adult brain tumors, whilst highlighting the parallel requirement for careful evaluation of unwanted toxicities prior to clinical trial consideration.

## Supporting Information

Figure S1 **No alterations in growth rate following non-acute RHPS4 exposure in PFSK-1 and C6 brain tumor cells.** (A) PFSK-1 cells were exposed to 0.2 µM RHPS4 for 23 days and (B) C6 cells were exposed to 2 µM RHPS4 for 36 days. No marked alteration in population doubling rate was observed for either cell line treated with RHPS4 concentrations ∼10-fold below IC_50_ concentrations during acute RHPS4 exposure (Figure 1).
(TIF)

Figure S2 **Brain tumor cells can proliferate upon RHPS4 removal.** Brain tumor cells were exposed to RHPS4 for 72 hours prior to removal of drug and continued culture for a further 48 hours in drug-free media. (A–B) PFSK-1 cells exhibit recovery of cells after removal of media containing 0.5, 1.0 and 2.0 µM RHPS4 and continued proliferation, whereas DAOY, C6 and GB-1 cells exhibit recovery of cells and continued proliferation after removal of each RHPS4 concentration. *Scale bar = 25* *µm*.
(TIF)
