# Supplementary figures and images for: RHPS4 G-Quadruplex Ligand Induces Anti-Proliferative Effects in Brain Tumor Cells
Source: PLoS One. 2014 Jan 15;9(1):e86187. doi: 10.1371/journal.pone.0086187 (PMC3893285; doi:10.1371/journal.pone.0086187)

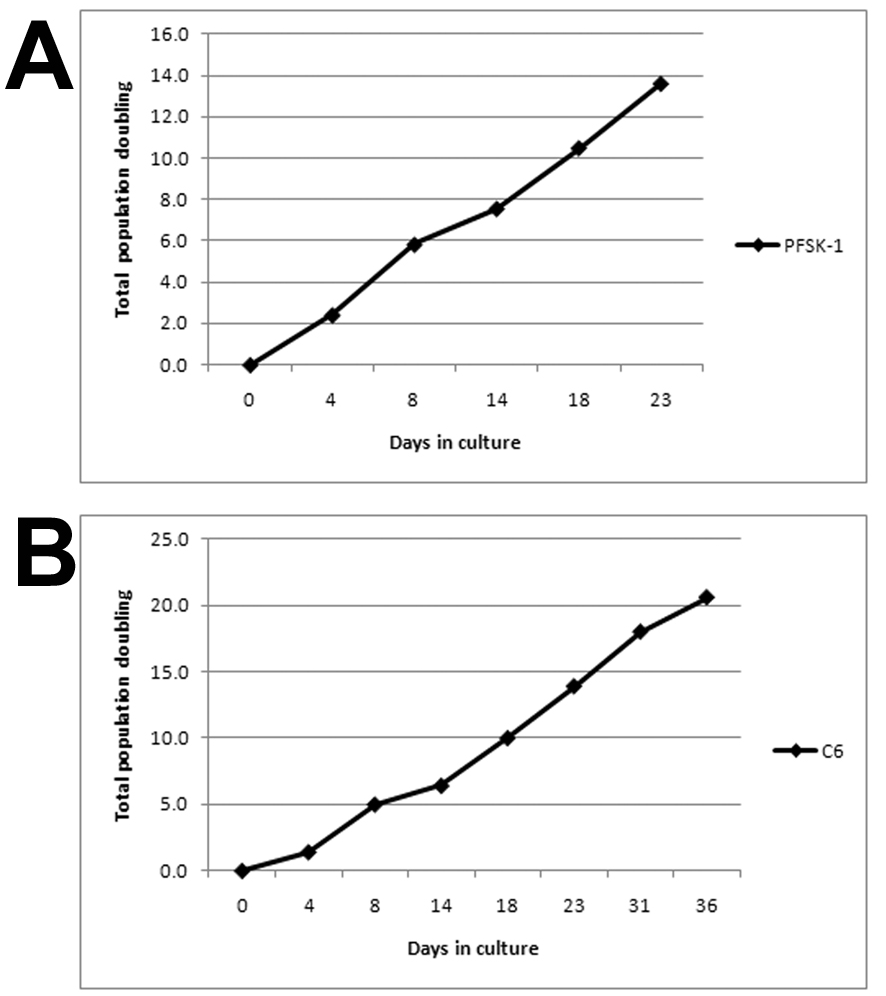

Supplement: Figure S1 — No alterations in growth rate following non-acute RHPS4 exposure in PFSK-1 and C6 brain tumor cells. (A) PFSK-1 cells were exposed to 0.2 µM RHPS4 for 23 days and (B) C6 cells were exposed to 2 µM RHPS4 for 36 days. No marked alteration in population doubling rate was observed for either cell line treated with RHPS4 concentrations ∼10-fold below IC50 concentrations during acute RHPS4 exposure (Figure 1). (TIF) [file pone.0086187.s001.tif]

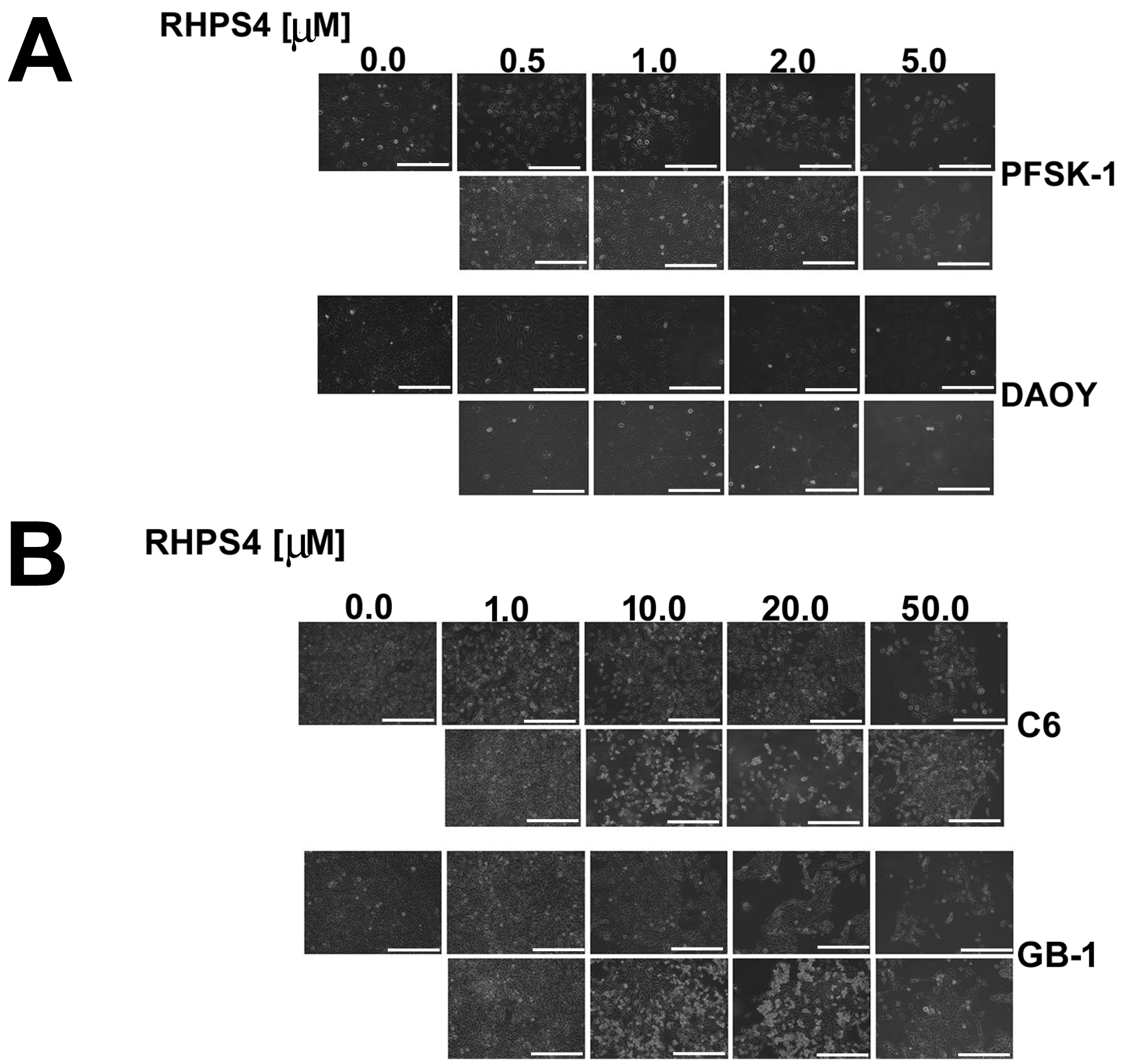

Supplement: Figure S2 — Brain tumor cells can proliferate upon RHPS4 removal. Brain tumor cells were exposed to RHPS4 for 72 hours prior to removal of drug and continued culture for a further 48 hours in drug-free media. (A–B) PFSK-1 cells exhibit recovery of cells after removal of media containing 0.5, 1.0 and 2.0 µM RHPS4 and continued proliferation, whereas DAOY, C6 and GB-1 cells exhibit recovery of cells and continued proliferation after removal of each RHPS4 concentration. Scale bar = 25 µm. (TIF) [file pone.0086187.s002.tif]
